# The in vitro effect of delta-9-tetrahydrocannabinol and cannabidiol on whole blood viscosity, elasticity and membrane integrity

**DOI:** 10.1186/s42238-022-00126-z

**Published:** 2022-04-05

**Authors:** Tameika R. James, Andrea A. Richards, Dwight A. Lowe, Walton A. Reid, Charah T. Watson, Dagogo J. Pepple

**Affiliations:** 1 Department of Basic Medical Sciences, Physiology Section, The University of the West Indies, Mona campus, Kingston, 7 Jamaica; 2 Department of Pathology, Haematology Section, University Hospital of the West Indies, Kingston, 7 Jamaica; 3 Electron Microscopy Unit, The University of the West Indies, Kingston, 7 Jamaica; 4 Bio-Tech R&D Institute, Kingston, Jamaica

**Keywords:** THC, CBD, Viscosity, Elasticity, Membrane integrity, Blood, Red blood cells

## Abstract

**Background:**

The main biological activities of cannabis are due to the presence of several compounds known as cannabinoids. Delta-9-tetrahydrocannabinol (THC) and cannabidiol (CBD) are two of the main cannabinoids. Studies have shown that the effects of THC can be modulated by CBD.

**Objective:**

This study aims to look at the effect of different concentrations of THC and CBD separately and in combination, on blood viscosity, elasticity and membrane integrity.

**Methods:**

Blood samples were collected from twenty-four healthy adult non-smokers. Blood viscosity and elasticity were determined using the Vilastic Scientific Bioprofiler for different concentrations (0, 2.5, 25, 50 and 100 ng/ml) of CBD and THC respectively, as well as in extracts with combinations of CBD and THC in 4:1 and 1:1 ratios respectively. Repeated measures analysis of variance (ANOVA) was used to determine the difference between the means of the groups.

**Results:**

Blood viscosity increased significantly with increasing concentrations of both THC and CBD from 25 ng/ml up to 100 ng/ml ranging from 6.45 ± 0.36 mPa·s to 11.60 ± 1.12 mPa·s for THC and ranging from 5.46 ± 0.24 mPa·s to 9.91 ± 1.10 mPa·s for CBD respectively, being more pronounced in the extracts at 21.33 ± 2.17 mPa·s for the 4THC:1CBD extract and 21.76 ± 1.88 mPa·s for the 1THC:1CBD extract. There was no significant increase in elasticity for THC and CBD separately. However, a significant increase in elasticity was observed in the extracts. THC and CBD affected red cell morphology resulting in complete disintegration at the highest concentrations.

**Conclusions:**

THC and CBD increased red blood cell viscosity and elasticity separately and in combination. They also adversely affected membrane integrity.

## Introduction

*Cannabis sativa* L. is widely distributed and grown throughout most of the temperate and tropical regions of the world. Cannabis is one of the chemically most complex plants due to the presence of a large number of compounds which can exert individual effects and may also interact with each other. Among these compounds, there are approximately one hundred and twenty C21 or C22 terpenophenolic compounds known as cannabinoids (Morales et al. 2017) which are considered to be the main biologically active constituents of the cannabis plant (Hazekamp et al. 2010; ElSohly and Slade 2005).

The cannabinoids belong to ten main subclasses of which five are the most abundant namely, Δ^9^-tetrahydrocannabinol (THC), cannabidiol (CBD), cannabigerol (CBG), cannabichromene (CBC) and cannabinol (CBN). THC has been found to be primarily responsible for the psychoactive properties of the cannabis plant (Hazekamp et al. 2010; Adams and Martin 1996). Lately, increasing attention has been focused on cannabidiol. Studies have shown that depending on the ratio in which they are administered, CBD may have either an antagonistic or potentiative effect on THC (Varvel et al. 2006).

Blood behaves as a viscoelastic material and exhibits both viscosity and elasticity properties (Thurston 1996). Viscosity refers to the energy dissipated during flow primarily due to sliding and deformation of red blood cells and red blood cell aggregates, while elasticity refers to the energy stored in the microstructure of blood during flow due to orientation and deformation of red blood cells (Thurston 1996). Increased blood viscosity has been linked to several disease conditions including sickle cell anaemia, cardiovascular disease, peripheral vascular disease, atherosclerosis, diabetes, stroke and other conditions (Baskurt et al. 2007). Both viscosity and elasticity are directly affected by red blood cell deformability which is itself affected by the mechanical properties (integrity) of the red blood cell membrane (Baskurt et al. 2007).

Cannabis is one of the most widely used illicit drugs worldwide (Goyal et al. 2017). There has also been increased legalization of cannabis for medicinal and recreational purposes (Alshaarawy and Elbaz 2016). There is a paucity of studies showing a direct link between cannabinoids and blood viscosity, elasticity and RBC morphology. While several studies have shown the effect of cannabis on the cardiovascular system (Latif and Garg 2020), they do not directly correlate the effects to blood viscosity, elasticity and RBC morphology in spite of the viscoelastic nature of blood and the dire consequences which can result from red blood cell dysfunction (Başkurt 2003). This study therefore provides further insight about the consequences of cannabis consumption.

There are several diseases in which abnormalities of blood viscosity, elasticity and RBC morphology may play a role. They include but are not limited to sickle cell anaemia, myocardial infarction, myocardial ischemia, peripheral artery disease, cerebral ischemia, diabetes and hereditary spherocytosis (Stoltz 1985). For the majority of these conditions, however, while the effect of blood viscosity and cannabis usage are known separately, there is no indication of how cannabis affects blood viscosity, and therefore, there is no indication about whether the effect of cannabis on these diseases are mediated through its effect on blood viscosity, while the same is true for sickle cell anaemia studies which have shown that vaso-occlusive crises occur more frequently in patients with high blood viscosity, and at least one study has shown that cannabis usage by persons with sickle cell anaemia resulted in more frequent hospitalizations due to the occurrence of vaso-occlusive crises (Ballas 2017), thereby providing an indirect link between blood viscosity, cannabis usage and sickle cell anaemia.

The aim of the study was therefore to look at the impact of THC and CBD on blood viscosity, elasticity and red blood cell membrane integrity which are all interdependent. Taking into consideration the fact that cannabis utilization is itself linked to several adverse outcomes to include arteriopathy, myocardial infarction and strokes and given its increasing utilization globally, it is of utmost importance to add to the body of knowledge surrounding the potential harmful effects of cannabis usage.

Additionally, since a search of the literature did not reveal any study that has directly linked the potential adverse effects of cannabis to its effect on blood viscosity, elasticity and membrane integrity and taking into consideration the increasing interest that currently surrounds the usage of cannabis and the development of medicinal products, it is of utmost importance to determine the effect of the constituents of cannabis on the parameters mentioned and therefore what impact they may have on the health of individuals who utilize cannabis. This study therefore aims to look at the effect of both THC and CBD separately and in combination on whole blood viscosity, elasticity and red blood cell membrane integrity.

## Materials and methods

### Subjects

Twenty-four non-smokers consisting of 14 females and ten males ranging in age from 21 to 42 years of age were recruited from the University of the West Indies, Mona Campus. They were recruited by speaking to staff and students of the Department of Basic Medical Sciences, Faculty of Medical Sciences, University of the West Indies, Mona Campus, about the research and soliciting their voluntary participation. No inducements were offered to solicit participation.

### Inclusion criteria

Participants had to be 18 years or older, be in good health and have normal haemoglobin (HbAA). Both male and female participants regardless of race were included in the study. All participants signed an informed consent form.

### Exclusion criteria

Participants were excluded if they smoked, utilized cannabis by any route of administration, had any circulatory diseases, did a blood transfusion recently and took or were currently using illicit drugs and persons who were currently on medication were not included in the study. The time frame for “currently” was not specified however, and participants were not required to state whether they used alcohol, tobacco or herbal products other than cannabis.

Informed consent was obtained from each participant before recruitment into the study. The study was approved by the UHWI/UWI/FMS Ethics Committee, UWI, Mona.

### Blood collection

Eight millilitres of venous blood was collected from the antecubital vein of each participant once and distributed into two vacutainer tubes containing K^+^EDTA (1.5 mg) as anticoagulant. Samples were kept at room temperature (25 °C) until measurements were done.

### Sample preparation

THC, CBD and extracts were obtained from Biotech R & D Institute, University of the West Indies, Mona Campus. Stock solutions were prepared using 5% ethanol.

0.1 mg of both THC and CBD were dissolved in 100 ml of ethanol to give stock concentrations of 1000 ng/ml. Aliquots of 2.5, 25, 50 and 100 μl were pipetted from each stock solution into Eppendorf tubes and then made up to 1 ml with blood to give concentrations of 2.5, 25, 50 and 100 ng/ml of THC and CBD respectively. A fifth Eppendorf tube was used as a control to which no THC or CBD was added.

Two extracts containing both THC and CBD in a 4:1 and 1:1 (THC:CBD) ratio were each also made up to 1 ml with blood in 5% ethanol to give a concentration of 50 ng/ml THC. Therefore, the extract with the 4:1 THC to CBD ratio contained 50 ng/ml THC and 12.5 ng/ml CBD while the extract with the 1:1 THC:CBD ratio contained 50 ng/ml THC and 50 ng/ml CBD. These concentrations were chosen based on concentrations of THC in plasma that were observed to give a psychoactive effect in previous studies (Watson et al. 2000).

On addition of the blood, the samples were gently agitated to allow for proper mixing and allowed to incubate for 20 min at room temperature (~ 25 °C) before measurements were taken. Blood samples were agitated by capping the tubes and inverting to mix as well as by rotation on an automated mechanical rocker (Sandrest Systems, Beeching Road, Bexhill, UK).

### Analysis of samples

The viscosity and elasticity of the blood samples were measured using the BioProfiler (Vilastic Scientific, Austin, TX, USA). The BioProfiler is capable of giving single measurements of viscosity, elasticity and relaxation time of whole blood at shear rates of 2.51 s^–1^, 12.6 s^–1^ and 62.8 s^–1^ and strain of 0.2, 1 and 5, respectively. Measurements were done at native haematocrit and at a frequency of 2 Hz, shear rate of 62.8 s^–1^ and strain of 5 at 37 °C.

The haematocrit was determined by the capillary tube method. The anticoagulated blood was centrifuged, after which a microhaematocrit reader (Vulcan Technologies, Missouri, USA) was used to determine the haematocrit based on the meniscus of the red blood cell layer.

Haemoglobin electrophoresis was performed on blood samples according to the method of Dacie & Lewis, 11th edition (Bain et al. 2012), to ascertain that all participants had normal haemoglobin genotype (HbAA).

The preparation of blood films was done according to the method of Dacie & Lewis, 11th edition (Bain et al. 2012). The slides were then stained with Wright’s stain following which they were visualized using a Nikon Eclipse E200 microscope (Nikon Instruments, Melville, NY, USA) under oil immersion at a magnification of × 100.

### Statistical analysis

The results were analysed using the IBM SPSS Statistics software version 20 and expressed as means ± standard error of the mean (SEM). Repeated measures analysis of variance (ANOVA) which incorporated the Bonferroni test was used to determine the difference between the means. Paired sample *T* test was also used where necessary to compare the difference in means between THC and CBD at similar concentrations and between the extracts and THC and CBD at 50 ng/ml. The statistical significance was taken at the 95% confidence interval, and a *p* value of < 0.05 was considered to be significant.

## Results

### Haemoglobin electrophoresis

All participants were found to have normal haemoglobin (HbAA) as determined by haemoglobin electrophoresis (Dacie & Lewis, 11th edition).

### Viscosity and elasticity

**Fig. 1:**
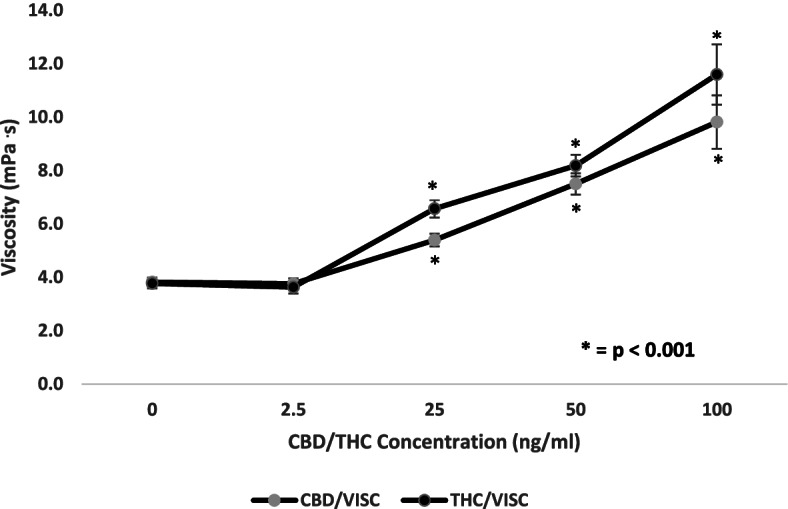
Comparison of the effect of increasing concentrations of delta-9-tetrahydrocannabinol (THC) and cannabidiol (CBD) (ng/ml) on blood viscosity (mPa·s). Blood was collected from the antecubital vein of 24 participants, and experiments were conducted in vitro. Values are expressed as means for plots (*n* = 24). Asterisk (*) indicates significant difference from control at *p* < 0.05. The error bars represent the standard error of each mean (CBD, cannabidiol; THC, delta-9-tetrahydrocannabinol)

The results indicate that there was a significant increase (< 0.05, 95% CI) in viscosity as the concentrations of THC and CBD increased from 25 ng/ml up to 100 ng/ml (3.77 ± 0.17 mPa·s for the control compared to 5.46 ± 0.24 mPa·s at 25 ng/ml, 7.51 ± 0.42 mPa·s at 50 ng/ml and 9.91 ± 1.10 mPa·s at 100 ng/ml for CBD in Fig. 1) (*F*(1.27, 25.47) = 27.39, *p* < 0.001) (3.74 ± 0.21 mPa·s for the control compared to 6.45 ± 0.36 mPa·s at 25 ng/ml, 8.07 ± 0.43 mPa·s at 50 ng/ml and 11.60 ± 1.12 mPa·s at 100 ng/ml for THC in Fig. 1) (*F*(1.36, 20.40) = 53.71, *p* < 0.001). The change in viscosity was not significant at the lowest concentration of 2.5 ng/ml. The results also indicate that the viscosity values were higher for THC than CBD at all concentrations except the lowest (Fig. 1). The viscosity values for THC however were only significantly higher than the values for CBD at 25 ng/ml (6.45 ± 0.36 mPa·s for THC and 5.46 ± 0.24 mPa·s for CBD) (*t*(17) = 5.57, *p* < 0.001) and 50 ng/ml (8.07 ± 0.43 mPa·s for THC and 7.51 ± 0.42 mPa·s for CBD) (*t*(17) = 2.34, *p* < 0.001).

**Fig. 2:**
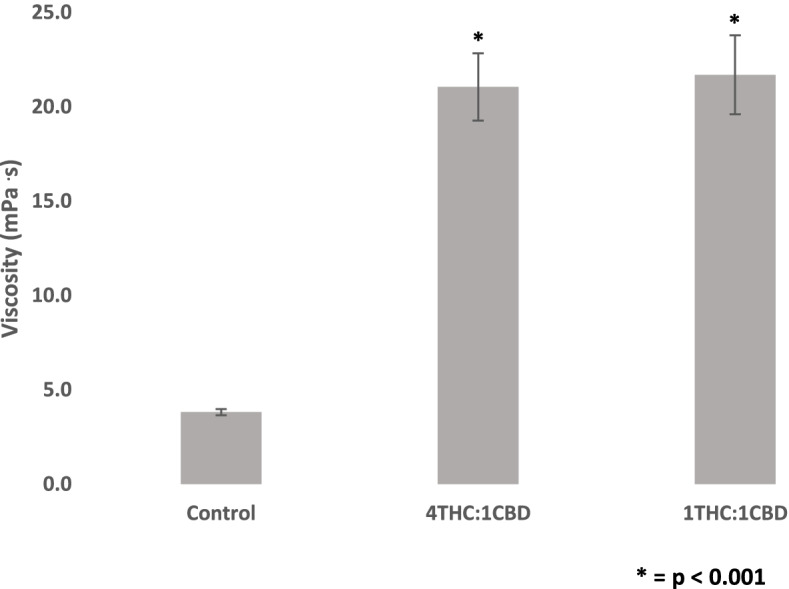
Change in blood viscosity (mPa·s) for two extracts containing different ratios of delta-9-tetrahydrocannabinol (THC) to cannabidiol (CBD) (4:1 and 1:1). Blood was collected from the antecubital vein of 24 participants, and experiments were conducted in vitro. Values are expressed as means for plots (*n* = 24). While the viscosity values for the 4THC:CBD and 1THC:1CBD extracts significantly differ from control, they are not significantly different from each other. Asterisk (*) indicates significant difference from control at *p* < 0.05. The error bars represent the standard error of each mean (CBD, cannabidiol; THC, delta-9-tetrahydrocannabinol)

There was a significant increase in viscosity for both Cannabis extracts that were used (21.33 ± 2.17 mPa·s for the 4THC:1CBD extract and 21.76 ± 1.88 mPa·s for the 1THC:1CBD extract as compared to control at 3.82 ± 0.81 mPa·s in Fig. 2) (*F*(2, 38) = 53.13, *p* < 0.001). This increase was even greater than the increases observed for THC and CBD separately (viscosity value for CBD at 50 ng/ml was 7.50 ± 1.87 mPa·s while the viscosity for THC at 50 ng/ml was 8.19 ± 1.72 mPa·s). The viscosity values for the extracts were significantly higher than the values obtained for THC and CBD at 50 ng/ml [(*t*(16) =7.20, *p* < 0.001) for THC and the 4THC:1CBD extract and (*t*(16) = 6.87, *p* < 0.001) for THC and the 1THC:1CBD extract] and [(*t*(20) = 8.06, *p* < 0.001) for CBD and the 4THC:1CBD extract and (*t*(20) = 7.55, *p* < 0.001) for CBD and the 1THC:1CBD extract]. There was however no significant difference in viscosity between the two THC:CBD extracts.

**Fig. 3:**
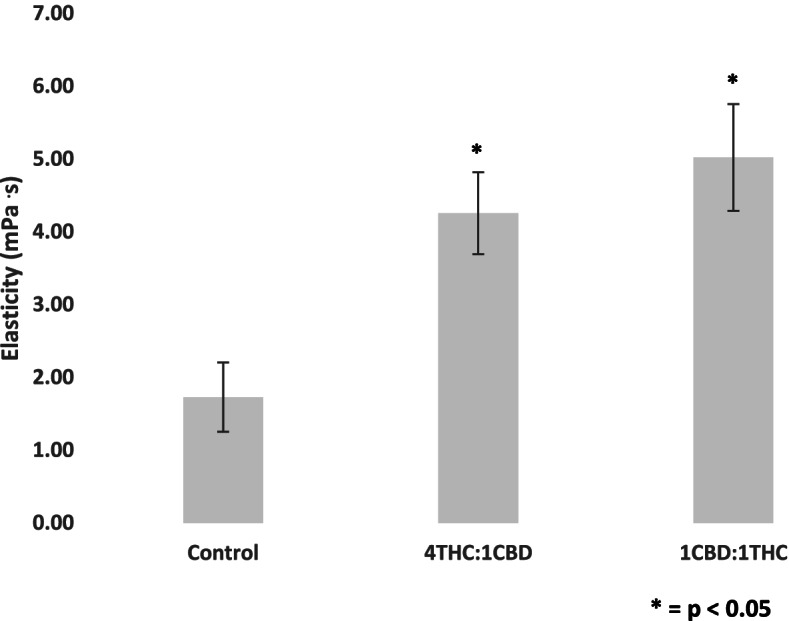
Comparison of the effect of increasing concentrations of delta-9-tetrahydrocannabinol (THC) and cannabidiol (CBD) (ng/ml) on blood elasticity (mPa·s). Blood was collected from the antecubital vein of 24 participants, and experiments were conducted in vitro. Values are expressed as means for plots (*n* = 24). The error bars represent the standard error of each mean (CBD, cannabidiol; THC, delta-9-tetrahydrocannabinol)

**Fig. 4:**
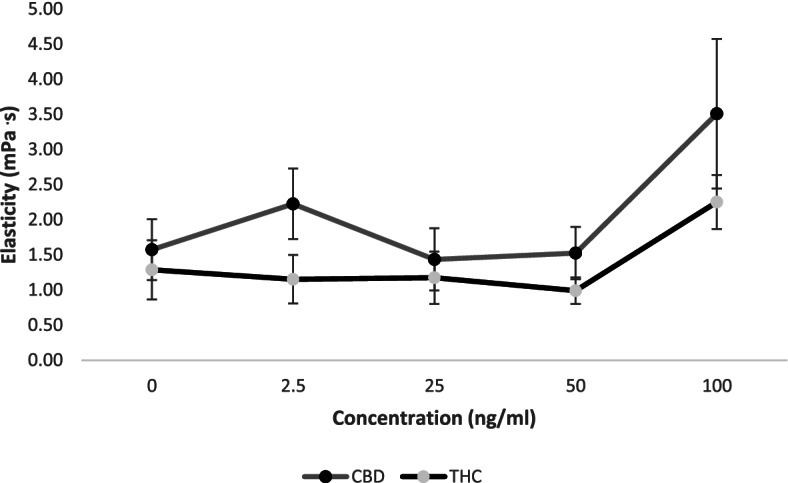
Change in elasticity (mPa·s) for two extracts containing different ratios of delta-9-tetrahydrocannabinol (THC) to cannabidiol (CBD) (4:1 and 1:1). Blood was collected from the antecubital vein of 24 participants, and experiments were conducted in vitro. Values are expressed as means for plots (*n* = 24). While the elasticity values for the 4THC:CBD and 1THC:1CBD extracts significantly differ from control, they are not significantly different from each other. Asterisk (*) indicates significant difference from control at *p* < 0.05. The error bars represent the standard error of each mean (CBD, cannabidiol; THC, delta-9-tetrahydrocannabinol)

When THC and CBD were used separately, there were no significant increases in elasticity (Fig. 3). Similarly, there was no significant difference in elasticity between the two THC:CBD extracts. The results however show a significant increase in elasticity for both extracts (4.41 ± 0.57 mPa .s for the 4THC:1CBD extract and 5.15 ± 0.76 mPa .s for the 1THC:1CBD extract as compared to control at 1.87 ± 0.51 mPa .s in Fig. 4); (F(1.53, 30.50) 7.88, *p* = 0.004).

### Blood films

**Fig. 5:**
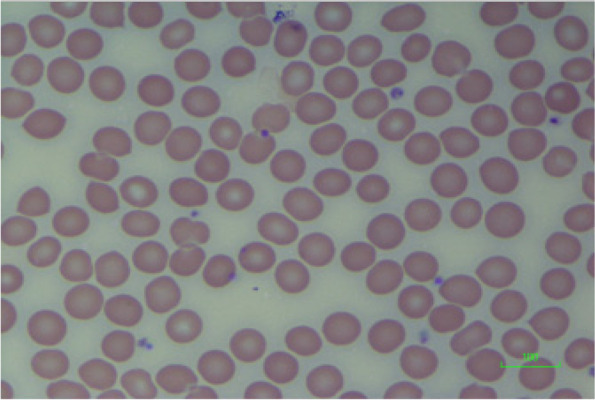
Blood film of control sample. Normal RBC morphology (control sample). Scale = 100 μm

In the film for the control sample (Fig. 5), the red blood cells appear as normal biconcave discs.

**Fig. 6:**
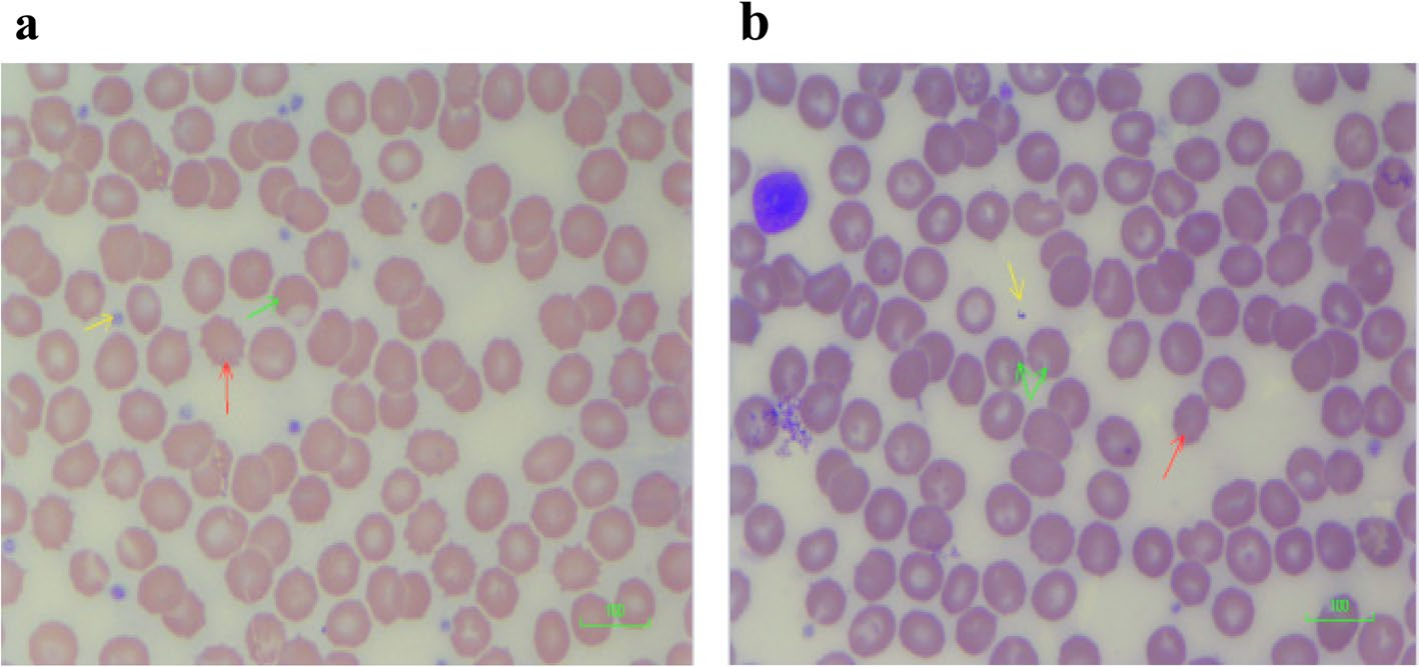
**a** Blood film of cells exposed to 50 ng/ml cannabidiol (CBD). Blood film showing red cell changes in 50 ng/ml CBD: burr cell formation (echinocytes). Scale = 100 μm (red arrow indicates burr cells; green arrow indicates blister cells, while the yellow arrow indicates extruded cytoplasm). **b** Blood film of cells exposed to 50 ng/ml delta-9-tetrahydrocannabinol (THC). Blood film showing red cell changes in 50 ng/ml THC: target cell formation, burr cells. Scale = 100 μm (red arrow indicates burr cells; green arrow indicates blister cells, while the yellow arrow indicates extruded cytoplasm)

Figure 6a shows the blood film for samples exposed to 50 ng/ml of CBD. The film shows the presence of burr cells. There is also some loss of cytoplasm as well as the presence of blister cells due to separation of haemoglobin from cell membranes. Similar to the cells exposed to 50 ng/ml CBD, analogous changes are seen in the cells exposed to 50 ng/ml of THC (Fig. 6b).

**Fig. 7:**
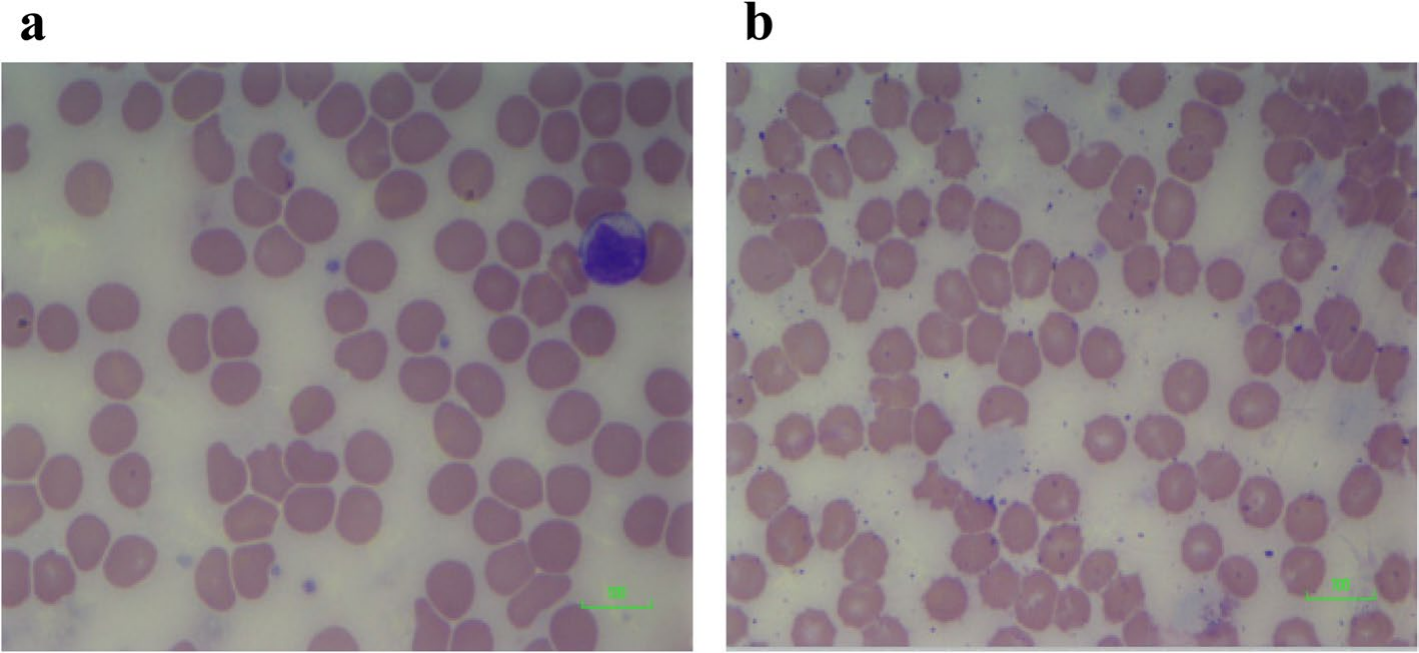
**a** Blood film of cells exposed to 100 ng/ml cannabidiol (CBD). Blood film showing red cell changes in 100 ng/ml CBD: red cell membrane distortion/indentation with occasional red cell fragments. Scale = 100 μm. **b** Blood film of cells exposed to 100 ng/ml delta-9-tetrahydrocannabinol (THC). Blood film showing red cell changes in 100 ng/ml THC: red cell fragmentation, burr cells. Scale = 100 μm

In addition to burr cells seen in Fig. 6a, Fig. 7a shows some amount of fragmentation and degeneration. These red blood cells were exposed to 100 ng/ml of CBD. The cells exposed to 100 ng/ml of THC (Fig. 7b) also showed similar changes to those observed in the cells exposed to 100 ng/ml of CBD.

**Fig. 8:**
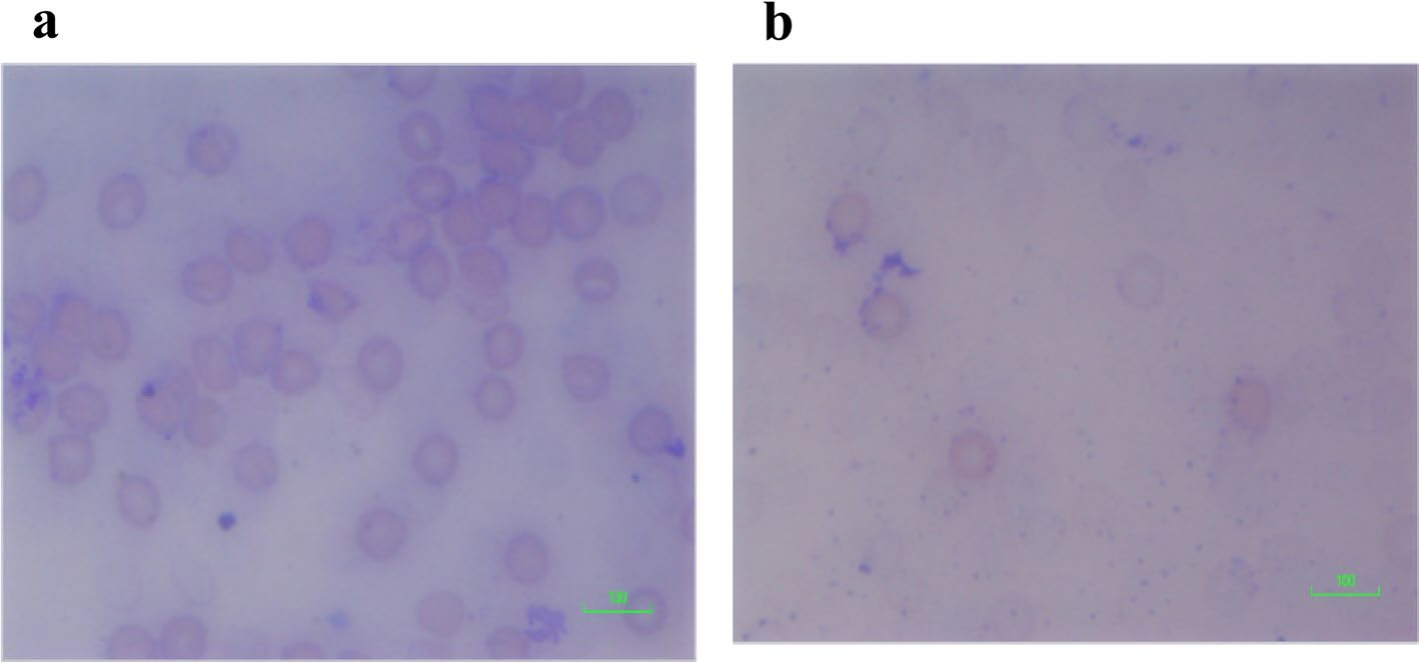
**a** Blood film of cells exposed to 1:1, THC:CBD extract. Blood film showing red cell changes in 1:1 THC:CBD extract—marked red cell agglutination with red cell degeneration. Scale = 100 μm. **b** Blood film of cells exposed to 4:1, THC:CBD extract. Blood film showing red cell changes in 4:1 THC:CBD extract—marked red cell degeneration; hardly any intact RBCs on film. Scale = 100 μm

Figure 8a shows marked red cell agglutination along with some RBC degeneration. These cells were exposed to the extract consisting of a one-to-one ratio of THC to CBD. For the cells exposed to a four to one ratio of THC to CBD (Fig. 8b), there was marked degeneration of red blood cells such that hardly any intact red blood cells were observed.

No results are presented for cells exposed to 2.5 ng/ml and 25 ng/ml of either THC or CBD. This is because these cells were similar in appearance to the control sample. Changes were observed at 50 ng/ml for both THC and CBD and progressively increased with increasing concentrations.

## Discussion

The results indicate that there is a significant effect of increasing concentrations of THC and CBD on blood viscosity. The observed increase is significant at all concentrations except at 2.5 ng/ml. This effect is even more pronounced with the extracts containing both THC and CBD. Two extracts were used with ratios of 4THC:1CBD and 1THC:1CBD. The viscosity values obtained for both extracts were significantly higher than the control sample, but they were not significantly different from each other indicating that regardless of ratio, THC and CBD have a stronger/potentiative effect on each other in relation to whole blood viscosity, when used in combination than when used separately.

The concentrations of THC and CBD used in the study were chosen based on concentrations of THC in plasma that were observed to give a psychoactive effect in previous studies (Watson et al. 2000) where it was shown that on administration of approximately 0.32 mg/kg (range: 0.22 to 0.50 mg/kg) of THC, the resulting plasma concentration ranged from 50 to 100 ng/ml with subjects indicating that they were approximately 50% high when the plasma concentration was 50 ng/ml. As these dosages were based on concentrations observed in the blood following the smoking of cannabis cigarettes with varying percentages of THC, taking into consideration that some THC may have been left in the butt or lost in the side stream smoke, they represent actual dosages that may be consumed by persons ingesting cannabis. The range of concentrations from 0 to100 ng/ml was therefore chosen to reflect the effect of THC and CBD both at low and high concentrations.

The fact that THC and CBD when used in combination have a stronger effect than when they are used individually is another example of the entourage effect which has also been observed in previous studies (Russo 2019). Studies have shown that depending on the ratio in which they are administered, CBD may have either an antagonistic or potentiative effect on THC (Varvel et al. 2006). In this study, however, there were no significant differences in the values obtained for the two extracts indicating that the difference in ratios used did not seem to play a role. The combined effect of THC and CBD was however found to be stronger than the individual effect.

The findings of the present study which indicates that blood viscosity increased with increasing cannabinoid concentrations are also indicative of a concomitant decreased deformability of the red blood cells as the concentrations of THC and CBD increased. This is supported by the results obtained for elasticity which though not significant when THC and CBD are used separately indicate that elasticity is highest when the highest concentration of either cannabinoid is used. The extracts, however, which contain different ratios of both cannabinoids show a significantly increased elasticity in comparison to the control used. Greater membrane elasticity indicates greater storage of elastic energy which therefore means that the red blood cell is more rigid and therefore less deformable as greater energy will be required in order for deformation to take place (Baskurt et al. 2007).

The cannabinoids are believed to exert their physiological effects primarily through two cannabinoid receptors which are referred to as CB1 and CB2 receptors. CB1 receptors are mainly expressed in the CNS, but they are also abundantly expressed in the PNS and other peripheral tissues and organs to include cardiac muscle, hepatic tissue, the gastrointestinal tract and vascular endothelium. The CB2 receptors on the other hand are primarily expressed in cells and tissues of the immune system although they are expressed to a lesser extent in the brain and other peripheral tissues (Subramaniam et al. 2019; Zou and Kumar 2018). Therefore, while cannabis has an effect on numerous systems throughout the body, increasing attention has been focussed on the adverse effects of cannabis on the cardiovascular system which include myocardial infarction, sudden death, peripheral arteritis and stroke (Wolff et al. 2015).

According to the literature, cannabis induces increased production of reactive oxygen species (ROS). This leads to increased oxidative stress which has been implicated in the occurrence of ischemic stroke and possibly other adverse cardiovascular events. It is believed that the production of the reactive oxygen species is primarily mediated by CB1 receptors (Han et al. 2009). The presence of reactive oxygen species can have deleterious effects on the proper functioning of erythrocytes whose main function in the circulatory system is the transport of oxygen and carbon dioxide to and from the lungs and tissues and also to maintain acid base equilibria. In this way, they play a very important role in cardiovascular homeostasis (Red blood cell function and dysfunction 2017). Increased production of ROS can have a profound impact on the integrity of the red blood cell membrane with the possibility of haemoglobin degradation, decreased red cell deformability and haemolysis, all of which have been implicated in serious pathological effects on the cardiovascular system.

One recent study (Ballas 2017) indicated that there was increased vaso-occlusive crises (VOC) in sickle cell patients who utilized cannabis resulting in more frequent hospitalizations. Based on the results obtained in this study, the observed increased incidence of VOC from that study was likely due to consumption of cannabis which could have led to increased viscosity, elasticity and impaired membrane integrity and therefore decreased erythrocyte deformability. Impaired erythrocyte deformability directly affects the ability of red blood cells to pass through small arterioles or capillaries (Baskurt and Meiselman 2003). The decreased deformability could further lead to blockage in the blood vessels, thereby resulting in increased VOCs. This can ultimately prevent oxygen delivery to the tissues and may result in hypoxia.

The increased viscosity and elasticity observed in this study indicate that as concentrations of THC and CBD in the blood increases, more energy is required for deformation and disaggregation of red blood cells. The red cells are therefore less likely to respond quickly to changes in circulation including the ability to change shape under stress imposed by high rates of flow through in the microcirculation. This will directly affect their ability to deliver oxygen to the tissues (Baskurt et al. 2007).

Since red cell deformability is influenced by three distinct cellular components namely cell shape geometry, viscosity of the cell cytoplasm and stability of the membrane (Baskurt et al. 2007; Lester et al. 2014), it would appear that THC and CBD are able to interact with these factors, thereby impairing red blood cell deformability and resulting in the increased viscosity and elasticity observed with increasing concentrations of both substances.

In conjunction with the increased viscosity and elasticity observed, there were increased morphological changes in the membrane of red blood cells with increasing concentrations of THC and CBD both separately and in combination. The results obtained therefore indicate that there is a dose dependent effect of THC and CBD on red blood cells. The cells were normal in appearance when 2.5 ng/ml and 25 ng/ml of THC and CBD were administered separately; however, at 50 ng/ml, there was evidence of impaired membrane integrity which worsened at 100 ng/ml. In the extract containing both THC and CBD in a 1:1 ratio, there was marked red cell agglutination along with degeneration, while in the extract containing THC and CBD in a 4:1 ratio, there was complete disintegration of red blood cells.

Red blood cells (RBCs) play a significant role in blood rheology due to the fact that they are the major cellular components of blood. As such, they are also the predominant factor that affects blood viscosity due to inherent characteristics of the red blood cells to include their ability to orient themselves with flow, haematocrit, the ability to elastically deform in response to mechanical forces and the capability to form rouleaux when there is low flow. Of note here is the fact that red blood cells are able to significantly increase blood viscosity when they lose their deformability which can significantly affect blood flow (Red blood cell function and dysfunction 2017).

While a search of the literature did not reveal specific studies linking RBC disintegration or haemolysis in human cannabis users with cannabis usage, studies have indicated that the psychological effects of THC were highest at the highest concentrations used in the study (Watson et al. 2000).

Changes in red cell morphology will affect the deformability of the red blood cells which in turn will affect their ability to enter microcirculation (Baskurt et al. 2007). The changes observed in red cell morphology therefore concur with the increased viscosity and elasticity observed with increasing concentration.

As previously mentioned, since there is a paucity of information in regard to the effect of cannabis on blood viscosity, elasticity and RBC morphology, this study helps to fill the gaps in knowledge that are not specifically covered in other studies. The results also give an indication that the lower concentrations of cannabinoids, more specifically THC and CBD may be safer and therefore more beneficial. Considering the increased utilization of cannabis for medicinal and recreational purposes, considering the increasing reports of adverse cardiovascular effects associated with cannabis use and considering the importance of blood viscosity, elasticity and RBC integrity in the maintenance of cardiovascular health, this study provides beneficial information which can help inform usage of cannabis.

The study was limited in that it was conducted in vitro rather than in vivo and therefore there is the possibility of variations arising in living systems. Limited quantities of reagents were also a limiting factor which prevented the conduction of the study on a wider scope such as the inclusion of a group of smokers for comparison purposes.

## Conclusion

Caution must be exercised in the consumption of cannabis products particularly in persons with sickle cell disease, as this could lead to decreased membrane deformability and increased haemolysis culminating in increased incidence of VOCs, painful crises and even stroke as well as worsening anaemia.

## Data Availability

The datasets used and/or analysed during the current study are available from the corresponding author on reasonable request.

## Abbreviations

THC: Delta-9-tetrahydrocannabinol
CBD: Cannabidiol
VOC: Vaso-occlusive crises
ROS: Reactive oxygen species
RBC: Red blood cell
